# Studying social media sentiment using human validated analysis

**DOI:** 10.1016/j.mex.2020.100867

**Published:** 2020-03-19

**Authors:** James Lappeman, Robyn Clark, Jordan Evans, Lara Sierra-Rubia, Patrick Gordon

**Affiliations:** a UCT Liberty Institute of Strategic Marketing, School of Management Studies, University of Cape Town, South Africa; b School of Management Studies, University of Cape Town, South Africa; c Senior Analyst, BrandsEye, South Africa; d Head of Marketing & Communications, BrandsEye, South Africa

**Keywords:** Social media, Negative word-of-mouth (nWOM), Online firestorms, Consumer sentiment

## Abstract

The measurement of online sentiment is a developing field in social science and big data research. The methodology from this study provides an analysis of online sentiment using a unique combination of NLP and human validation techniques in order to create net sentiment scores and categorise topics of online conversation. The study focused on measuring the online sentiment of South Africa's major banks (covering almost the entire retail banking industry) over a 12-month period. Through this methodology, firms are able to track shifts in online sentiment (including extreme firestorms) as well as to monitor relevant conversation topics. To date, no published methodology combines the use of big data NLP and human validation in such a structured way.

• *Microsampling for manual validation of sentiment analysis (both qualitative and quantitative approaches in order to obtain the most accurate results)*
• *Sentiment measurement*
• *Sentiment map*

## Graphical abstract

Key:A. Net sentiment in June dropped to -35.7% due to backlash over a controversial Absa Twitter poll. B. Net sentiment increased to over 30% during June and July amid news that Capitec was nearing the 10 million client mark. C. Net sentiment drops to -50.0% in December due to clients complaining about unauthorised debit orders. D. -16.1% net sentiment in December over issues with payments and branch experiences. E. Standard Bank's systems were temporarily offline, driving net sentiment down to -26.0% in February. The graphical abstract shows the net sentiment for each bank over the 12 month period. The key provides certain notable peaks and dips in net sentiment and the reasons for these shifts.

**Figure:**
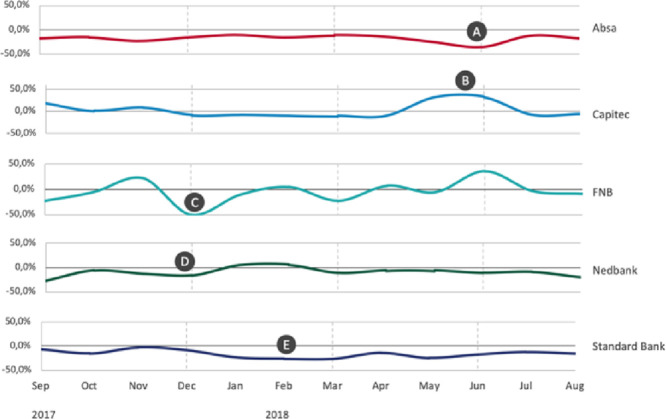

**Table:** Specifications Table

Subject Area:	Online behaviour
More-specific subject area:	*Social media sentiment*
Method name:	*Online sentiment analysis with human validation*

## Method details

The nature of the study set out was rather complex and relatively new, based on prior research examined; thus, specific considerations were taken into account in order to design the methodology. Collaboration from an external company, BrandsEye, was required in order to help analyse mass data from the banking industry [3]. A predominant number of the consumer research studies found are reliant on backwards-looking indicators, such as questionnaires or focus groups [23], [26], [14]. For this study, however, social data were examined and allowed for unsolicited and non-coercive responses that captured the lived experiences of consumers within the banking industry [29].

In more recent years, content analysis has been a means by which social media has been analysed [13], [21], [25]. Content analysis is usually done by means of a mixed-method approach of both qualitative and quantitative methods [4]. The qualitative approach is mainly inductive, exploring the underlying meaning of messages as well as drawing inferences from the data (comments/posts), whereas quantitative content analysis is deductive, using probabilistic approaches to test hypotheses generated from either theories or previous empirical studies performed [30]. Typical sentiment analysis utilises NLP (natural language processing) only approaches, which in the absence of human assistance often is unable to accurately evaluate the nuances of human conversation [17]. In this study, NLP was used for initial relevancy determination in order to help ensure that all mentions harvested were relevant to the research question(s). The process then combined a human validation (or crowdsourcing) in order to increase the precision of the data [17]. Hence, this study used a combination of both qualitative (using human validation through topic analysis) and quantitative (using a combined process of sentiment analysis and topic analysis) approaches in order to obtain the most accurate results.

### Data collection and sampling

The first step was to mine and analyse social media posts from the selected social media platforms (Facebook, Instagram, YouTube, LinkedIn and Twitter). Conversations surrounding the retail banks were accessed via an application programming interface (API), by which a filter was used to collect the relevant posts mentioning any of South Africa's top five retail banks over the designated period of a year (1 September 2017 to 31 August 2018). GNIP (a social media API aggregation company) collects social media data from various platforms and subsequently normalises the data [28] to be sent to clients. GNIP provided this study access to data in order to conduct the necessary research. Through this, a relative analysis of online consumer sentiment regarding selected conversations was acquired [28]. A major advantage of using the GNIP platform is gaining an accurate, real-time market opinion of the relationship between consumers and the retail banks in question. From here, a machine learning algorithm, discussed below, was used to evaluate each of the posts and their sentiments towards each bank [2].

Through this process, algorithms sifted through millions of posts and retrieved 1 720 810 posts containing mentions of the retail banks in question from the selected social media networks. These 1 720 810 posts made up the study's sampling population. These posts have come from a randomly selected group of individuals who have publicly posted on social media, mentioning any of the relevant topics regarding South Africa's top five retail banks (Absa, Capitec, FNB, Nedbank and Standard Bank). Geocoding was not analysed. The high use and continuous escalation of major social media platforms by both consumers and brands [11], as well as the limited number of studies which extensively focus on multiple social media platforms, corroborate the appropriateness of using multiple social media platforms for this study [32]. A non-probability technique was adapted through using quota sampling, whereby individuals were chosen by their social media posts retaining relevant topics.

### Measuring sentiment

Sentiment analysis is a computer process developed to identify and categorise consumers’ opinions online in order to determine their sentiment towards a particular issue (a specific bank in this case) [12]. The sample of social media posts that were collected then underwent a sentiment analysis. This involved categorising the text in each post as either positive or negative sentiment, posts with neutral sentiment being disregarded [1]. All enterprise posts were excluded from the analysis in order to prevent bias and maintain a focus on the consumer segment. Traditionally when analysing sentiment, there are two approaches (machine learning algorithms and a lexicon approach) that can be adopted to measure and scale the contents within each post. While both approaches are seen to have similar accuracy in analysing sentiment applied to consumer-generated content [7], this research made use of the machine learning algorithm approach, following other researchers in the field [16], [27], [33]. Due to the nuances of human conversation, certain entries could not be accurately processed by artificial intelligence; thus human insight was required in order to ensure accuracy [8], [24]. Entries were tagged with their sentiment, finding whether positive or negative emotions evoked from it ([2], [1], [22].) This process of including human involvement is the process of *manual validation,* whereby a sub-sample of the data was analysed by verified human contributors (micro-sampling for manual validation), which will be further discussed in the next section.

### Micro-sampling for manual validation

Micro-sampling via manual validation can also be considered as a reliability assessment ([20]). Within this process, a sub-sample of 521 326 randomly selected posts from the various platforms were selected and individually analysed by human ‘raters’ (or human contributors). These qualified contributors rate the data for relevancy, sentiment and topics. Each mention is sent to multiple raters’ for verification and is based on a consensus model that accounts for conflicting ratings. Inter-rater reliability is measured through challenges, appeals and judgements on mentions that are processed. Approximately 0.02% of mentions were challenged or appealed, suggesting a high degree of inter-rater reliability. Contributors were pre-trained in the process and also required to have locally relevant knowledge on the topic of conversation and a thorough understanding of the English language. The contributors were remunerated in micropayments for executing these micro jobs.

The use of human validation was critical to this study due to the fact that the complex and emotive nature of conversations (using social nuances, such as slang or sarcasm) are less easily picked up in analyses by algorithm-based systems [12], [31]. Thus, by using manual validation with the team of human contributors, this research study was able to examine the nuances and meaning behind a significant number of posts, ensuring that the data were correctly categorised. This has ensured that all major themes and sentiments driving consumers opinions have been uncovered, even when they have not been clearly stated or implied. The table below (Table 1) illustrates the sampling rates of data collected across the various social media platforms during the sampling period, providing specific inferences to the number of posts that went through the human validation process as well as the topic analysis process.

**Table 1:** Sampling Rates.

Bank	Volume of mentions	Crowd verification	Topic analysis	Margin of error
Absa	417 500	108 179	24 569	0.26%
Capitec	326 825	98 473	38 504	0.26%
FNB	493 885	147 097	54 302	0.21%
Nedbank	238 240	89 208	21,183	0.26%
Standard Bank	244 630	78 369	21 264	0.28%

In Table 1, the topic analysis column refers to the sample of sentiment-verified mentions that were sent for assignment to topics. The margin of error column refers to how many percentage points this calculation's result will differ from the real population value. For example, a 95% confidence interval with a 4% margin of error means that the statistic will be within 4 percentage points of the real population value 95% of the time. In the case of the banking data that was verified for sentiment, the margin of error for each bank ranged from 0.21% to 0.28% for the margin of error. The combined process of the machine learning algorithm and manual validation has created a confidence level of 95% and an overall 0.1% margin of error. Through the presented methodology, this shows the reliability and validity of the data.

### Topic analysis

In order to determine which topics and specific issues were driving consumer sentiment in the study, a topic analysis was conducted. Topic analysis enables a more in-depth understanding of the themes which drive consumer sentiment, and enables researchers to categorise these drivers in order to draw specific inferences from large collections of data [15]. The topic analysis was conducted utilising a natural language processing (NLP) technique (whereby non-verbal conversations can be understood). Here, keywords or related topics were generated by the human contributors or programmer, which allowed for any relevant posts to be selected for the sample population [15]. In the NLP process the study made use of Mallet (McCullum, 2002), which is maximum entropy classifier trained on labelled data (from the topic analysis) to classify mentions according to relevance to the brand/organisation. This has allowed researchers to understand not only how consumers feel, but also the key issues that are driving consumers to feel a certain way [3].

Seventy defined banking topics were chosen prior to the data analysis, through extracting all the different topics conversed about amongst both positive and negative social media posts. The topics were then categorised by human contributors and machine learning algorithms into seven umbrella topics (themes). The predefined themes were general themes that were derived from reading through the literature on the banking industry, professional definitions, local common-sense constructs and human contributors’ own values and prior experiences. This method is supported in prior studies, as by the time that these themes can be applied to the text, a great deal of interpretive analysis has been done [18], [5], [6].

**Fig. 1:**
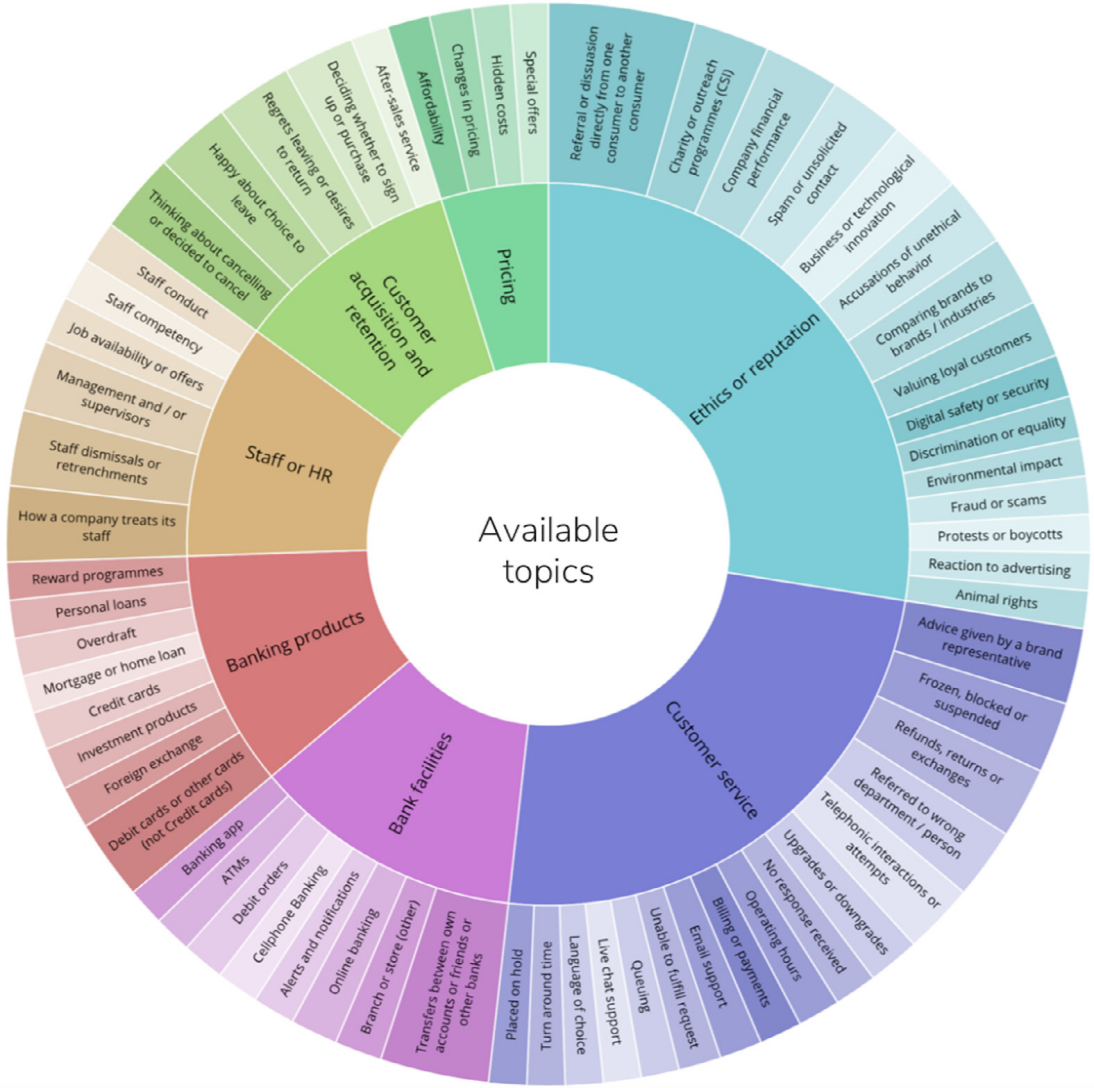
Topic wheel illustrating the 7 broad themes and 70 banking topics.

These themes were generated through having analysed a sub-sample of 159 822 posts, whereby the content was grouped by a thematic analysis based on the topic at hand, and were subsequently grouped into broad thematic clusters. This form of topic analysis develops more precise outcomes when compared to other topic-analysis methods, such as topic modelling [10]. A closer look at the themes and topics can be seen in Fig. 1.

### Analysis procedure

The analysis procedure was conducted in two fundamental components: analysing the individual banks, followed by analysing the industry as a whole. This unique process was necessary due to the complex nature of this study.

Within the first component of the analysis procedure, each individual bank's sentiment was investigated throughout the study period to get a measure of net sentiment. Net sentiment was calculated by deducting negative sentiment from positive sentiment. Weighted net sentiment was calculated by multiplying net sentiment by the total conversation volume. Specific focus was made to inferences of spikes in negative sentiment which indicated the presence of online firestorms. The term, ‘online firestorm’ is defined by Pfeffer, Zorbach and Carley in 2014 as “the sudden discharge of large quantities of messages containing negative WOM and complaint behaviour against a person, company, or group in social media networks” [34]. The analysis paid particular attention to which factors (identified themes and topics) caused a downturn in sentiment, as well as taking note of when negative sentiment climaxed during a firestorm. The fact that the net sentiment could be calculated over the year period allowed a view of sentiment over time. When a downward curve was detected in the sentiment score (a firestorm), a deeper analysis was possible into the specific factors that drove the change. Specifically, when a specific company's net sentiment would deviate from the aggregated industry net sentiment, causes could be identified. This was done while monitoring the duration of the decline from its starting point to any form of resolution or dissolution of negative sentiment. Finally, the brands content performance was analysed, focusing on volume, engagement and average engagement per post over the period. The volume refers to brand content (whilst excluding enterprise reshares, direct messages and replies and automated brand posts), effectively being the number of posts made by the individual bank within the duration period. The engagement score was limited to consumer engagement with the brand, this being the number of times consumers engaged with any post within the volume.

The overall analysis of the banks focused on three parts. Within the first part, it investigated a topic analysis. The topic analysis explored broad conversation themes identified within the banking industry, as well as the overall sentiment within those themes. The main negative themes for each bank were also analysed. Following this, a response analysis was conducted. This focused on looking at response rates on the social media platform Twitter, average brand response times and finally the overall sentiment of final interactions with the individual banks. These data consisted of posts from consumers who expressed dissatisfaction with verified negative posts, inclusive of the specific bank's name. The final part of the analysis investigated and compared the net sentiment across all of the banks. This section explored the share of voice across the banks, providing a visual comparison of the volume of social media interaction achieved by these five banks and finally analysed net sentiment and how the trends changed after time.

## Ethical considerations

This research has used a covert approach, as the respondents were not aware that their posts were being recorded for research purposes. This can propose ethical implications due to the fact that it infringes on people's awareness of how their online content can be used. Disclosed in the terms and conditions of social media, public posts made by individuals grant the world a non-exclusive licence to use, process and display content, effectively legalising the use of shared social media posts ([9]). All data from privatised accounts are considered restricted and were not used (Pak & Parouki, 2010). No direct messages between consumers and company handles were used in the analysis. Within the human validation process, any data indicating that the user would not wish to share their opinions were considered and the data were not used. All respondents made use of within the study remain completely anonymous.

## Methodological contribution

The methodology explained in this article is an early contributor to the combined use of both computer and human analysis of social media sentiment analysis for a study spanning a full year. Through the ability to accurately analyse unsolicited consumer sentiment data, insights have been found into how consumers feel about their bank (the subject of this particular study). The value of this longitudinal source of unsolicited human feedback can assist in making improvements in a person or organisation's reputation, risk, market conduct and service. Sentiment analysis provides researchers with the ability to gather unsolicited, reliable and cheaper data than those of surveys and polls [19]. The accuracy and reliability are due to sentiment analysis's access to vast social networking services, giving researchers further access to a larger sample. The population generalisability, while hard to quantify in the social sciences, would be far greater using this scale of research when compared to traditional survey data.
